# Responses of Three Successive Generations of Beet Armyworm, *Spodoptera exigua,* Fed Exclusively on Different Levels of Gossypol in Cotton Leaves

**DOI:** 10.1673/031.010.14125

**Published:** 2010-09-29

**Authors:** Gang Wu, Jian-Ying Guo, Fang-Hao Wan, Neng-Wen Xiao

**Affiliations:** ^1^ State Key Laboratory for Biology of Plant Diseases and Insect Pests, Institute of Plant Protection, Chinese Academy of Agricultural Sciences, Beijing 100081, PR China; ^2^ College of Plant Sciences and Technology, Huazhong Agricultural University, Wuhan 430070, China; ^3^ Chinese Research Academy of Environmental Sciences, No.8 Dayangfang Anwai, Chaoyang, Beijing 100012, PR China

**Keywords:** food utilization, secondary compounds

## Abstract

The beet armyworm, *Spodoptera exigua* (Hübner) (Lepidoptera: Noctuidae), is an important pest of numerous crops, and it causes economic damage in China. Use of secondary metabolic compounds in plants is an important method used to control this insect as a part of integrated pest management. In this study the growth, development, and food utilization of three successive generations of *S. exigua* fed on three cotton gossypol cultivars were examined. Significantly longer larval life-spans were observed in *S. exigua* fed on high gossypol cultivar M9101 compared with those fed on two low gossypol cultivars, ZMS13 and HZ401. The pupal weight of the first generation was significantly lower than that of the latter two generations fed on ZMS13 group. Significantly lower fecundity was observed in the second and third generations of *S. exigua* fed on M9101 compared with *S. exigua* fed on ZMS13 and HZ401. The efficiency of conversion was significantly higher in the first and third generations fed on HZ401 compared with those fed on ZMS13 and M9101. A significantly lower relative growth rate was observed in the three successive generations fed on M9101 compared with those fed on ZMS13 and HZ401. Cotton cultivars significantly affected the growth, development, and food utilization indices of *S. exigua,* except for frass and approximate digestibility. Development of *S. exigua* was significantly affected by relative consumption rate and efficiency of conversion of ingested food, but not by relative growth rate or approximate digestibility, suggesting that diet-utilization efficiency was different based on food quality and generation. Measuring the development and food utilization of *S. exigua* at the individual and population levels over more than one generation provided more meaningful predictions of long-term population dynamics.

## Introduction

The beet armyworm, *Spodoptera exigua* (Hübner) (Lepidoptera: Noctuidae) is an important pest of numerous crops, and it causes economic damage in China. Historically, *S. exigua* has been managed as part of the control of pests of cotton, *Gossypium hirsutum* L. (Malvales: Malvaceae), and initially received little attention following the adoption of control procedures for cotton varieties in China. The failure of chemical measures to control this insect has shifted the emphasis toward effective implementation of an integrated pest management (IPM) program.

Cotton cultivars with high gossypol levels are considered to be resistant to herbivorous insects and have been adopted by cotton growers for the purpose of herbivorous insects' management (Gao et al. 2008). Gossypol, a phenolic sesquiterpenoid aldehyde, is an important allelochemical occurring in gossypol glands of cotton cultivars. This allelochemical exhibits antibiosis to many pests and contributes to the host plant resistance of cotton varieties with gossypol glands (Zhou 1991). Du et al. (2004) and Gao et al. (2008) have indicated that high gossypol levels in the cotton plant had negative effects on *Aphis gossypii* and produced a positive effect on growth and development of *Propylaea japonica* at the third trophic level. Chen et al. (1997a, 1997b) reported that wheat plants with high levels of resistance to aphids had high amounts of plenolics and tannin. Wu et al. (unpublished data) found that different cotton gossypol levels significantly affected the enzyme activities in *S. exigua.* Other researchers showed a relationship between gossypol level and herbivorous insects' population abundance (Bottger et al. 1964; Meng and Li 1999).

Most published studies of responses of herbivorous insects to botanical secondary substances have been short-term experiments (e.g., one generation, or a certain instar) measuring development and consumption rates (Du et al. 2004; Gao et al. 2008). However, experiments conducted over more than one generation revealed differences in responses between generations and also showed that the outcome could depend on conditions of host-plant growth (Brooks and Whittaker 1998, 1999; Wu et al. 2006). Despite the well-recognized need for additional long-term studies on this topic (Lindroth et al. 1995; Wu et al. 2008), few studies have evaluated the development and food utilization of herbivorous insects over multiple generations (Wu et al. 2007a). Wu et al. (2006) reported that significantly longer larval life-span for the third generation and lower pupal weight for all generations were observed in *Helicoverpa armigera* fed on milky grains of spring wheat grown in elevated CO_2_. The performance of the leaf beetle, *Gastrophysa viridula,* was slightly affected by elevated CO_2_ after three consecutive generations fed on *Rumex* plants, despite measurable declines in indices of foliage quality and small eggs at the end of the second generation, which led to fewer and smaller larvae in the third generation (Brooks et al. 1998). Chen et al. (2007) reported an increased cotton bollworm larval lifespan, food consumption rate, relative consumption rate (RCR), and approximate digestibility (AD) of transgenic Bt cotton. Results from the above experiments show that further multigenerational studies are needed to predict the development and consumption rates of herbivorous insects at individual and population levels.

As a serious pest of cotton, *S. exigua* was added to a list of out-break insect pests in 2001 in China (Shi et al., 2006). Despite the recognized need for long-term studies, few studies published to date have been conducted for more than one generation to evaluate population dynamics (Wu et al. 2007a). In the present study, three successive generations of *S. exigua* fed on three cotton cultivars were reared, and the objective was to evaluate the cumulative effect of gossypol on the development, food utilization, and population performance of *S. exigua* over three successive generations.

## Materials and Methods

### Cotton variety and growth condition

The three cotton cultivars used in the study included ZMS13, HZ401, and M9101 with gossypol contents of 0.06%, 0.44%, and 1.12%, respectively (Du et al. 2004; Gao et al. 2008). The three cotton cultivars were planted in plastic pots (15 cm diameter, 13 cm height) in a climate controlled chamber. The temperature was maintained at 28 ± 1° C, and relative humidity was maintained at 70–80%. For each cotton cultivar, 120 pots were randomly placed in the chamber and re-randomized once a week to minimize position effects. Soil pH was 7.1, organic matter 14.5%, available N 397.7 mg Kg^-1^ (hydrolic N, 1 N NaOH hydrolysis), available P 269.0 mg Kg^-1^ (0.5 *M* NaHCO_3_ extraction), and available K 262.2 mg Kg^-1^ (1 N CH_3_COONH_4_ extraction). Water (200 ml) was added to each pot once every three days after cotton seedling emergence. No chemical fertilizers or insecticides were used throughout the experiment.

### Insect feeding

The egg masses of & *exigua* were obtained from the Insect Virology Laboratory, Institute of Zoology, Chinese Academy of Sciences and hatched in a growth chamber ((PRX-500D-30; Haishu Safe Apparatus, http://nbhssfsy.cn.china.cn/). The chamber was maintained at 75 ± 5% RH, 28 ± 0.5° C, and 14:10 L:D at 30,000 LX of active radiation supplied by 39 26-W fluorescent lamps in the chamber.

During the three cotton cultivars' 8–10 leaf stage (≈ 40–50 d after planting), leaves were gathered and supplied as food to neonate larvae kept in the same chamber temperature, RH, and photoperiod conditions as detailed above. Twenty insects were reared individually in a glass dish (75 mm in diameter) with four replications per treatment. Ten leaves were randomly selected with three replicates for each cotton cultivar, oven dried at 80° C for 72 h, and used to calculate the proportion of dry matter to water content immediately prior to the beginning of the insect rearing trials. Simultaneously, fresh leaves with petioles were provided daily to *S. exigua* larvae. Each day, the frass and remaining portion of cotton leaves were collected and oven dried at 80° C for 72 h. The fresh body weights were measured every other day. Larval development was calculated as the period from hatch to pupation. Pupal weight was measured ∼12 h after pupation was noted, and the rate of pupation was recorded. For each treatment, survival rate was calculated as the number of moths emerged divided by the number of first instar larvae.

*S. exigua* were sexed and recorded to calculate the proportion of females among the total number of adults after emergence. The emerging moths were enclosed in a cage (30 × 30 × 40 cm) for two days to achieve mating, and then paired (one female and one male) in a plastic cup (9 cm in diameter; height of 15 cm) with a net cover of absorbent cotton yarn for oviposition. The numbers of eggs laid in each cup were counted daily, and the cotton yarn was replaced daily. The eggs were tracked for each female and kept in artificial climate incubators (PRX-500D-30; Haishu Safe Apparatus, www.nbhssfsy.cn.china.cn) to record hatching. Eighty neonates from each generation were followed through the complete life cycle for three successive generations following the same rearing protocol as the first generation.

### Foliar chemical compositions assays of cotton plants

Comparable leaves to those in the feeding studies were randomly collected at the same time, placed in liquid nitrogen for 3 h, and then transferred to a -20° C refrigerator for later use in chemical composition assays. Five leaves with three replicates were taken for each cotton cultivar. Leaf water content, as a proportion of fresh weight, was calculated after drying at 80° C for 72 h. Protein, total amino acids, and free fatty acids were assayed according to manufacturer's instructions (Nanjing Jiancheng Ltd. Co., www.njjcbio.com). Nitrogen content was measured using a CNH analyzer (Model ANCA-nt; Europa Elemental Instruments).

### Development, consumption, and food utilization indices of *S. exigua*

**Growth and development indices.** Four indices were used to measure the growth and development of *S. exigua* including larval development, pupal weight, survival rate, and fecundity.

**Indices for larval consumption and utilization.** The conventional, ratio-based nutritional indices, including relative growth rate (RGR, mg/g/day), relative consumption rate (RCR, mg/g /day), efficiency of conversion of ingested food (ECI, %), and approximate digestibility (AD), were determined gravimetrically following the methods of Waldbauer (1968) and Scriber and Slansky (1981). The amount (mg) of food ingested, frass produced, larval body weight, and weight gain were all calculated as dry weights. Formulae for calculation of the indices are shown in Chen et al. (2005).

### Data Analysis

One-way ANOVAs (SAS 6.12, SAS Institute Inc. USA, 1996) were used to analyze the foliar chemical compositions of the three cotton cultivars. Population indices, consumption, and frass were analyzed using two-way ANOVAs with cotton cultivar and *S. exigua* generations as sources of variability, where the cotton cultivar was the main factor and *S. exigua* generation was a sub-factor deployed in a split-plot design. The data for larval consumption and digestibility indices were analyzed using an analysis of covariance (ANCOVA) with initial weight as a covariate for RCR, RGR, ECI, and AD. Food consumption was a covariate for ECI to correct for the effect of variation in the growth and food assimilated on intake and growth (Raubenheimer and Simpson 1992); and food assimilated was also used as a covariate to analyze the ECD parameter (Hägele and Martin 1999). The assumption of a parallel slope between covariate and dependent variables was satisfied for each analysis. Means were separated using the least significant difference (LSD).

## Results

### Foliar chemical composition assays of cotton plants

Significantly lower foliar nitrogen content (p < 0.001), protein content (p < 0.05), and free fatty acid content (p < 0.001) were observed in the high gossypol cultivar, M9101, compared with the two low gossypol cultivars, ZMS13 and HZ401. However, foliar water content (p > 0.05) and total amino acids (p > 0.05) were not significantly different between the three cotton cultivars (Table 1).

### Growth and development of three successive generations of *S. exigua*

**Larval life-span and pupal weight.** Cotton cultivar significantly affected larval life-span (p < 0.0001) and pupal weight (p < 0.0001). *S. exigua* generation also significantly influenced larval life-span (p < 0.01) and pupal weight (p < 0.01). The interaction between cotton cultivar and *S. exigua* generation significantly affected pupal weight (p < 0.01) (Table 2).

There was a difference in larval life-span in the first (*F* = 4.87; df = 2, 9; p = 0.04), second (*F* = 27.56; df = 2, 9; p = 0.0001), and third (*F* = 19.35; df = 2, 9; p = 0.001) generations of *S. exigua* fed on M9101 compared with *S. exigua* fed on ZMS13 and HZ401. The larval life-span of the third generation was significantly longer than that of the previous two generations fed on the low gossypol cultivar, ZMS13 (*F* = 6.35; df = 2, 9; p = 0.02). There was a difference in pupal weight in the second (*F* = 15.11; df = 2, 9; p = 0.001) and third (*F* = 16.30; df = 2, 9; p = 0.001) generations of & *exigua* fed on ZMS13 compared with *S. exigua* fed on M9101 and HZ401. The pupal weight of the first generation was significantly lower than that of the previous two generations fed on low gossypol cultivar ZMS13 (*F* = 17.65; df = 2, 9; p = 0.001) (Table 3).

**Table 1:**
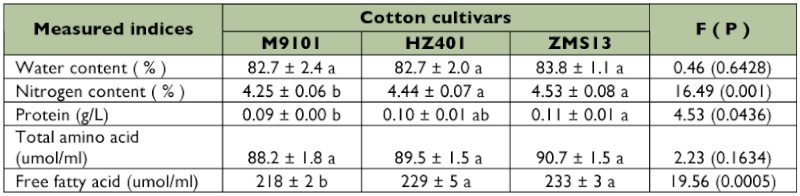
Contents of chemical compositions (Mean ± SD) in the leaves of three cotton cultivars.

**Table 2:**
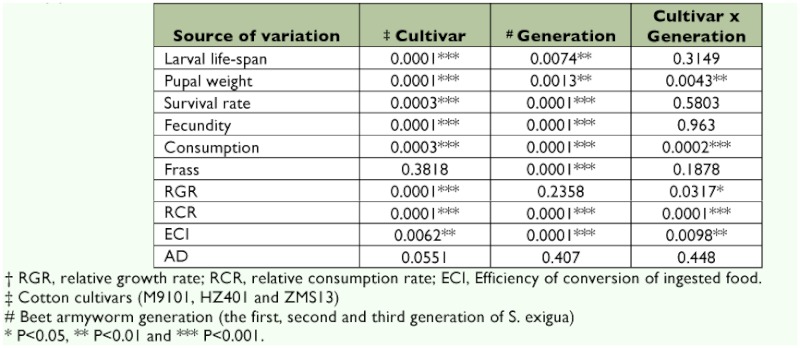
Effects of cotton cultivar, beet armyworm generation and cotton cultivar × beet armyworm generation on the life history parameters and food utilization of beet armyworm, *S. exigua.*

**Survival rate and fecundity.** Cotton cultivars significantly affected survival rate (p < 0.001) and fecundity (p < 0.0001). *S. exigua* generation also significantly influenced survival rate (p < 0.0001) and fecundity (p < 0.0001) (Table 2).

There was a difference in the survival rate of the third generation (*F* = 9.33; df = 2, 9; p = 0.01) of *S. exigua* fed on ZMS13 compared with those fed on M9101 and HZ401. The survival rate was found to be significantly different among the three successive generations of *S. exigua* fed on M9101 (*F* = 22.2; df = 2, 9; p = 0.0003), HZ401 (*F* = 36.3; df = 2, 9; p = 0.0001), and ZMS13 (*F* = 48.9; df = 2, 9; p = 0.0001). There was a difference in fecundity in the second (*F* = 7.43; df = 2, 9; p = 0.01) and third (*F* = 5.59; df = 2, 9; p = 0.03) generations of & *exigua* fed on M9101 compared with those fed on ZMS13 and HZ401. The fecundity was significantly decreased for the first generation compared to that of the latter two generations fed on M9101 (*F* = 13.4; df = 2, 9; p = 0.002), HZ401 (*F* = 9.36; df = 2, 9; p = 0.01), and ZMS13 (*F* = 13.76; df = 2, 9; p = 0.002) (Table 3).

### Consumption and food utilization of three successive generations of *S. exigua*

**Consumption and frass.** Cotton cultivar significantly affected consumption per larva (p < 0.001). Successive generation significantly influenced the consumption and frass per larva (p < 0.0001). The interaction between cotton cultivar and generation significantly affected consumption per larva (p < 0.001) (Table 2).

**Table 3:**
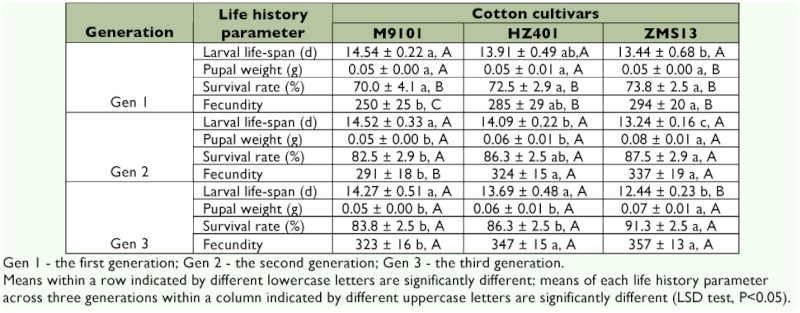
Mean ± SD of life history parameters of three successive generations of Beet Armyworm, *S.* *exigua* (Hübner) fed on three cotton cultivars.

**Figure 1.**
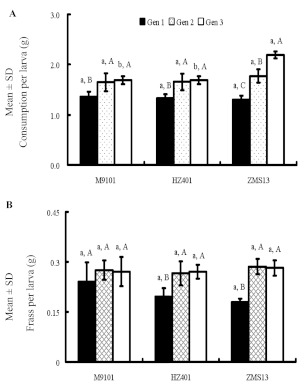
Consumption (A) and frass (B) of three successive generations of beet armyworm, *Spodoptera exigua,* fed on three cotton cultivars. (Gen 1 - the first generation; Gen 2 - the second generation; Gen 3 - the third generation; Different lowercases show significant differences among three cotton cultivars. Different uppercases indicate significant differences across three generations by LSD tests at p < 0.05). High quality figures are available online.

There was a difference in consumption per larva in the third generation (*F* = 57.43; df = 2, 9; p = 0.0001) of *S. exigua* fed on ZMS13 compared with those fed on M9101 and HZ401. The consumption per larva was significantly decreased in the first generation compared to that of the latter two generations fed on M9101 (*F* = 8.35; df = 2, 9; p = 0.0089), HZ401 (*F* = 11.73; df = 2, 9; p = 0.003), and ZMS13 (*F* = 80.88; df = 2, 9; p = 0.0001) (Figure 1A). There was a difference in frass per larva in the first generation compared to that of the latter two generations fed on HZ401 (*F* = 8.77; df = 2, 9; p = 0.0077) and ZMS13 (*F* = 38.02; df = 2, 9; p = 0.0001) (Figure 1B).

### Indices for larval utilization

**RGR and RCR.** Cotton cultivar significantly affected the relative growth rate (RGR, mg/g/day) and relative consumption rate (RCR, mg/g/day) of *S. exigua* (p < 0.0001). *S. exigua* generation significantly influenced the RCR (p < 0.0001). The interaction between cotton cultivar and generation significantly affected the RGR (p < 0.05) and RCR of *S. exigua* (p < 0.0001) (Table 2).

There was a difference in RGR in the first (*F* = 15.41; df = 2, 9; p = 0.001), second (*F* = 15.19; df = 2, 9; p = 0.001), and third (*F* = 53.07; df = 2, 9; p = 0.0001) generations of *S. exigua* fed on M9101 compared with those fed on ZMS13 and HZ401. However, significantly higher RGR was found in the third generation than that of the previous two generations when fed on ZMS13 (*F* = 8.34; df = 2, 9; p = 0.009). There was a difference in RCR in the third generation (*F* = 162.27; df = 2, 9; p = 0.0001) of *S. exigua* fed on M9101 compared with those fed on ZMS13 and HZ401. The RCR of *S. exigua* was significantly decreased in the first generation compared to that of the latter two generations fed on M9101 (*F* = 7.15; df = 2, 9; p = 0.01), HZ401 (*F* = 9.32; df = 2, 9; p = 0.01), and ZMS13 (*F* = 108.56; df = 2, 9; p = 0.0001) (Table 4).

**ECI and AD.** Cotton cultivar significantly affected the efficiency of conversion of ingested food (ECI, %) (p < 0.01). *S. exigua* generation significantly influenced the ECI (p < 0.0001). The interaction between cotton cultivar and *S. exigua* generation significantly affected the ECI (p < 0.01) (Table 2).

There was a difference in ECI in the first (*F* = 7.83; df = 2, 9; p = 0.01) and third (*F* = 11.29; df = 2, 9; p = 0.004) generations of *S. exigua* fed on HZ401 compared with those fed on ZMS13 and M9101. The ECI *of S. exigua* was significantly increased in the first generation compared to that of the latter two generations fed on M9101 (*F* = 11.98; df = 2, 9; p = 0.003), HZ401(*F* = 8.48; df = 2, 9; p = 0.01), and ZMS13 (*F* = 89.32; df = 2, 9; p = 0.0001). There was a difference in AD in the second generation compared with the first and third generations (*F* = 10.08; df = 2, 9; p = 0.005) fed on ZMS13 (Table 4).

## Discussion

Secondary metabolic compounds of plants are an important biochemical basis for plant resistance to insects. Using plant resistance to insects is a major method for controlling insect pests in modern integrated pest management (Cai et al. 2004; Wu et al. 2007b). Gossypol produced by cotton is one of the most important toxic chemicals to herbivorous insects and is a major source of modern biological insecticide, which is considered one of the key insect resistance mechanisms. Du et al. (2004) reported that high gossypol in host cotton had an negative effect on *A. gossypii* and showed a positive effect on growth and development of *P. japonica* at the third trophic level. Stipanovic et al. (2008) found that higher gossypol concentrations were required to reduce survival and pupal weights and increase days-to-pupation for larvae of *Heliothis virescens* larvae compared with the concentration needed to affect larvae of *H. zea* (Boddie). Most of these initial published studies focused on responses of herbivorous insects to gossypol in short-term experiments. Combining short- and long-term experiment findings can provide a clearer picture of insect population dynamics in response to gossypol.

**Table 4:**
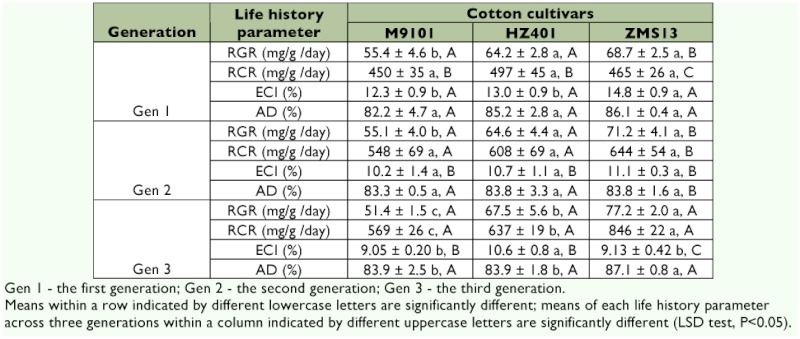
Mean ± SD of indices of three successive generations for of beet armyworm, *S. exigua* fed on three cotton cultivars.

In the present experiments, larval development was increased by 4.54% and 8.18% in the first generation, 3.09% and 9.69% in the second generation, and 4.27% and 14.73% in the third generation after feeding on the high gossypol cultivar M9101 compared with those fed on ZMS13 and HZ401. These results showed that direct negative effects of secondary metabolic plant compounds (e.g., gossypol in this study) were observed on *S. exigua.* The pupal weight and survival rate were not significantly different in the first generation of *S. exigua* after feeding on the three cotton gossypol cultivars. However, pupal weight was significantly increased by 42.4% in the second generation and by 37.9% in the third generation after feeding on the low gossypol cultivar, ZMS13, compared with those fed on the high gossypol cultivar, M9101. Also, the survival rate significantly increased by 6.06% in the second generation and 8.96% in the third generation after feeding on the low gossypol cultivar, ZMS13, compared with those fed on the high gossypol cultivar M9101. The results showed that *S. exigua* can develop significant resistance or tolerance to secondary metabolic plant compounds (e.g., gossypol in this study) under continuous selection pressure through three successive generations.

Plant nitrogen is known to be an important element for insect success (Mattson 1980), and reduction in food protein and nitrogen content can often lead to poorer insect performance, or behavioral or physiological adaptation (Scriber and Slansky 1981). Insects feeding on low nitrogen foliage exhibit reduced larval growth (Roth et al. 1994, 1995), increased foliage consumption (Williams et al. 1994), and reduced fecundity (Traw et al. 1996). Most leaf-chewing insects exhibit compensatory increases in food consumption (Scriber 1982). These results are generally explained as a response of herbivorous insects to reduced forage quality, especially the reduction in forage nitrogen (Wu et al. 2006). Most of the initial published studies focused on food compensate for response of herbivorous insects in short-term experiments (Wu et al. 2008). However, the emergence of combining short- and long-term experiments has provided a clearer picture of the dynamics (Brooks et al. 1998).

In this study, significantly lower relative growth rates (RGR) were found in three successive generations of *S. exigua* fed on the high gossypol cultivar, M9101, compared with those fed on the low gossypol cultivar, ZMS13. However, the efficiency of conversion of ingested food (ECI) was only significantly decreased in the first generation fed on M9101 compared with those fed on ZMS13. It is likely that the reduction of RGR is due to cumulative effects of secondary metabolic compounds (presumably gossypol in this study) on three successive generations of *S. exigua.* The results showed food quality on the diet-utilization efficiency of herbivorous insects was different, along with the insect species and insect stages. Other published documents strengthen this standpoint (Wu et al. 2006). Brooks and Whittaker (1998) reported the ECI of the leaf beetle, *Gastrophysa viridula,* was significantly reduced by elevated CO_2_, but RGR was significantly reduced in the second generation and was increased by elevated CO_2_ in the third generation. RGR of third instars was not affected by elevated CO_2_ in any generation. Wu et al. (2008) observed that the RGR was significantly reduced in three successive generations of *S. exigua* fed on transgenic Bt cotton compared with those fed on non-transgenic Bt cotton. However, the relative consumption rate (RCR) significantly decreased only in the first generation of *S. exigua* fed on transgenic Bt cotton compared with those fed on non-transgenic Bt cotton.

The results of this experiment provide a much clearer understanding of the direct effects of the secondary metabolic compound gossypol, on *S. exigua.* The experiment attempted to determine responses of *S. exigua* through different developmental stages and generations. Measuring the development and food utilization of *S. exigua* at the individual and the population level over more than one generation provides more meaningful predictions of long-term population dynamics. Development and implementation of multigenerational pest management tactics has become critical to ensuring the long-term efficiency of cotton cultivars in monitoring the field population dynamics of *S. exigua.*

## Abbreviations

AD: approximate digestibility;
ECI: efficiency of conversion of ingested food (%);
RCR: relative consumption rate (mg/g/day);
RGR: relative growth rate (mg/g/day)
